# A randomized phase I trial of nanoparticle albumin-bound paclitaxel with or without mifepristone for advanced breast cancer

**DOI:** 10.1186/s40064-016-2457-1

**Published:** 2016-06-30

**Authors:** Rita Nanda, Erica M. Stringer-Reasor, Poornima Saha, Masha Kocherginsky, Jean Gibson, Bernadette Libao, Philip C. Hoffman, Elias Obeid, Douglas E. Merkel, Galina Khramtsova, Maxwell Skor, Thomas Krausz, Ronald N. Cohen, Mark J. Ratain, Gini F. Fleming, Suzanne D. Conzen

**Affiliations:** Section of Hematology/Oncology, Department of Medicine, The University of Chicago, 5841 S. Maryland Ave., MC 2115, Chicago, IL 60637 USA; Department of Health Studies, The University of Chicago, 5841 S. Maryland Ave., MC 2000, Chicago, IL 60637 USA; Northshore University Health Systems, 2650 Ridge Ave # 4805, Evanston, IL 60201 USA; Department of Pathology, The University of Chicago, 5841 S Maryland Ave., MC 6101, Chicago, IL USA; Section of Endocrinology, Department of Medicine, The University of Chicago, 5841 S. Maryland Ave., MC 1027, Chicago, IL 60637 USA; 5841 S. Maryland Ave., MC 2115, Chicago, IL 60637-1470 USA

## Abstract

**Purpose:**

Glucocorticoid receptor (GR) overexpression is associated with poor prognosis ER-negative breast cancer. GR antagonism with mifepristone increases chemotherapy-induced breast cancer cell death, therefore we conducted a phase I clinical trial of mifepristone and nab-paclitaxel in advanced breast cancer.

**Methods:**

A novel randomized phase I design was used to assess the effect of mifepristone on nab-paclitaxel pharmacokinetics and toxicity. Patients were randomized to placebo or mifepristone for the first cycle; mifepristone was given to all for subsequent cycles.

**Results:**

Nine patients were enrolled. All were found to have a twofold or greater increase in serum cortisol after mifepristone administration, reflecting effective GR inhibition. Neutropenia occurred at both nab-paclitaxel dose levels studied (100 and 80 mg/m^2^), and was easily managed with dose reduction and/or growth factor administration. Pharmacokinetic data suggest an interaction between nab-paclitaxel and mifepristone in some patients. Two patients had complete responses (CR), three partial responses (PR), one stable disease (SD), and three progressive disease (PD). Immunohistochemical staining for GR found six of nine tumors were GR-positive. All six GR-positive tumors were triple-negative at the time of recurrence. Of these six patients, two had CRs, two PRs, one SD, and one PD.

**Conclusions:**

GR appears to be a promising target in TNBC, and GR inhibition plus chemotherapy produces manageable toxicity. While neutropenia was observed in some, a nab-paclitaxel dose of 100 mg/m^2^ plus mifepristone 300 mg was found to be tolerable, and a randomized phase II trial of nab-paclitaxel with/without mifepristone is planned in GR-positive advanced TNBC.

**Electronic supplementary material:**

The online version of this article (doi:10.1186/s40064-016-2457-1) contains supplementary material, which is available to authorized users.

## Background

The glucocorticoid receptor (GR) is expressed in a significant subset of human breast cancers (Conzen 2008). In ER-negative breast cancer, but not ER-positive breast cancer, high GR expression in the primary tumor is associated with a significantly higher risk of relapse (Pan et al. 2011). In vitro and in vivo experiments suggest that activation of the GR in ER-negative pre-malignant breast epithelial and cancer cells initiates cell survival pathways under otherwise apoptosis-inducing conditions (e.g. chemotherapy, radiation, and growth factor deprivation) (Wu et al. 2004). Glucocorticoid-mediated GR activation is associated with cancer cell resistance in preclinical models by activating the expression of genes whose protein products significantly inhibit chemotherapy-induced apoptosis (Skor et al. 2013). We hypothesize that GR antagonism will enhance chemotherapy sensitivity of GR+/ER− breast cancer cells by blocking stress-mediated cell survival pathways that would otherwise counteract chemotherapy-induced apoptosis in tumor cells.

Mifepristone is a potent glucocorticoid receptor (GR) and progesterone receptor (PR) antagonist, as well as a weak androgen receptor (AR) antagonist (Song et al. 2004). Mifepristone is currently FDA approved for the treatment of hyperglycemia secondary to Cushing’s disease and termination of pregnancy (Spitz and Bardin 1993; Johanssen and Allolio 2007). While a single dose of 200 mg of mifepristone combined with misoprostol is sufficient to terminate pregnancy, studies in Cushing’s Syndrome suggest that higher doses may be required for potent anti-GR effects (Nieman et al. 1985). Animal studies suggest that GR antagonism may be of value in the treatment of a variety of diseases such as glucocorticoid-dependent hypertension, arthritis, glaucoma, psychosis, and addiction, although clinical studies have yet to be reported. Several small single agent studies of mifepristone and another PR antagonist onapristone have been evaluated for advanced breast cancer with disappointing results. However, these studies have been focused on use of these agents as PR antagonists in PR positive disease (Klijn et al. 2000; Romieu et al. 1987; Perrault et al. 1996; Bakker et al. 1990).

Taxanes and anthracyclines remain among the most active and widely used chemotherapy agents used to treat breast cancer in the adjuvant as well as metastatic setting (Vishnu and Roy 2011). Paclitaxel inhibits mitosis and leads to cell death by binding to dimerictubulin and causing disruption of microtubule disassembly. Response rates for paclitaxel in taxane naïve patients with metastatic breast cancer have ranged from 20 to 60 %. Weekly therapy appears to be more efficacious and has less hematologic toxicity than every-3-week dosing (Seidman et al. 2008). One major limitation of paclitaxel, however, is its poor water solubility. Due to poor solubility, paclitaxel must be dissolved in the solvent Cremophor. Cremophor is associated with many side effects, including anaphylaxis, and requires premedication with glucocorticoids (Shepherd 2003).

Nab-paclitaxel is an albumin-bound, solvent-free novel formulation of paclitaxel that eliminates the need for premedication with glucocorticoids. A large phase II study evaluating weekly nab-paclitaxel (at a dose of 100 mg/m^2^ given weekly for 3 weeks out of 4) demonstrated response rates of 14–16 % in taxane-resistant, previously treated metastatic breast cancer patients (Blum et al. 2007). While nab-paclitaxel is an effective and well-tolerated therapy for MBC, many tumors do not respond to therapy, and even those that do initially respond eventually go on to develop resistance.

We hypothesized that GR antagonism with mifepristone prior to the administration of cytotoxic chemotherapy will improve efficacy by blocking the strong anti-apoptotic signal mediated by GR activation via circulating endogenous cortisol. Here we present the first clinical trial combining a glucocorticoid receptor (GR) antagonist with nab-paclitaxel. Nab-paclitaxel was used instead of paclitaxel, as it does not require glucocorticoid premedication, which would counter the GR antagonism of mifepristone. As paclitaxel (and therefore nab-paclitaxel) is a known CYP2C8 substrate, and mifepristone is a known inhibitor of CYP2C8, co-administration of the two drugs had the potential to increase paclitaxel levels. We therefore utilized a randomized phase I design to assess the impact of mifepristone on paclitaxel pharmacokinetics and toxicity. The purpose of the trial was to study the safety and tolerability of the combination and determine the recommended phase II dose of the combination of mifepristone plus nab-paclitaxel.

## Methods

This study was conducted at The University of Chicago Medicine (Chicago, IL) and Northshore University HealthSystem (Evanston, IL), and was approved by the respective Institutional Review Boards. Written informed consent was obtained prior to any study procedures. The study was registered on ClinicalTrials.gov as NCT01493310.

### Eligibility criteria

Patients were eligible for this study if they had metastatic or locally advanced breast cancer not amenable to local therapy, were ≥18 years of age, had an ECOG PS ≤ 2. Measurable disease was not required. Patients with ER and/or PR positive disease must have progressed on at least one prior hormonal therapy. Patients were required to have normal organ and marrow function.

### Exclusion criteria

Patients were excluded from this study if they had a history of allergy or hypersensitivity to mifepristone, paclitaxel, or drugs of similar chemical composition, had received more than four prior cytotoxic therapies for metastatic disease, or prior nab-paclitaxel or mifepristone for metastatic disease. Patient who were pregnant or breast feeding, had peripheral neuropathy > grade 1, had long term or concurrent use of corticosteroid therapy, or had significant uncontrolled intercurrent illness were also excluded. A washout period of 4 weeks was required before initiation of therapy.

### Drug supply

Nab-paclitaxel was obtained commercially in single-dose vials (manufactured by Celgene). Mifepristone (300 mg tablets) and matching placebo tablets were supplied by Corcept Therapeutics.

### Study treatment

Treatment was administered on an outpatient basis. No concomitant investigational or commercial agents or therapies administered with the intent to treat the patient’s malignancy were allowed, with the exception of bisphosphonates (e.g. zoledronic acid) and RANKL inhibitors (e.g. denosumab) for patients with bone metastases.

Patients were randomized to nab-paclitaxel plus mifepristone versus nab-paclitaxel plus placebo treatment during the first cycle of each dose level in a 3:2 ratio (with a planned minimum of five patients per dose level). Treatment assignment was double-blinded to ensure that stress (which might elevate serum cortisol levels) between treatment group was equivalent, and to ensure unbiased assessment of adverse events attributable to treatment. Only patients randomized to mifepristone were used for toxicity assessment and determination of mifepristone dose escalation. Patients were unblinded at the end of cycle 1, and placebo patients were crossed over to mifepristone treatment at the current dose level beginning in cycle 2.

Patients were treated with intravenous weekly nab-paclitaxel on day 1, 8 and 15 of each 28 day cycle at a starting dose of 100 mg/m^2^. Mifepristone was administered orally for two consecutive days, starting one day prior to each nab-paclitaxel infusion at a starting dose of 300 mg per day.

### Dose escalation/de-escalation

A novel randomized phase I design was utilized, although the mifepristone dose escalation was to follow the traditional ‘3 + 3’ design with up to four dose cohorts (300, 600, 900, and 1200 mg) to determine the maximally tolerated dose (MTD). The starting dose of mifepristone was 300 mg. Toxicity was assessed weekly during the first cycle of therapy and on day 1 of cycle 2. Adverse events were assessed among all patients, but only mifepristone patients’ adverse events during cycle 1 were used for the purposes of dose-escalation decisions.

The nab-paclitaxel starting dose was 100 mg/m^2^ (dose level 1), with the plan to de-escalate to 80 mg/m^2^ (dose level −1) and 60 mg/m^2^ (dose level −2) for subsequent dose levels as needed.

### Dose-limiting toxicity (DLT)

Dose-limiting toxicity was defined as any grade III toxicity not reversible to grade II or less within 96 h, or any grade IV toxicity (excluding nausea and vomiting). Toxicity was graded according to CTCAE version 4.0. A patient who received any amount of drug was considered evaluable for toxicity. However, patients who did not receive at least five of the six doses of mifepristone and all of the three planned doses of nab-paclitaxel during cycle 1 for reasons other than toxicity/tolerability, were considered inevaluable for determination of MTD and were replaced. A dose reduction, omission, or delay of dose for toxicity performed during cycle 1 or on day 1 of cycle 2 constituted a DLT.

### Study assessments

The patient’s medical history was taken at baseline. A physical examination, ECOG status assessment, complete blood count with differential, and a comprehensive metabolic panel were conducted at baseline and weekly for 3 weeks in a row with 1 week off though out the treatment period. All adverse events and laboratory abnormalities were assessed at baseline and during treatment. Antitumor activity was evaluated every 2 cycles (8 weeks).

### Pharmacokinetic analyses

Paclitaxel concentrations were determined on cycle 1, days 2 and 9 for all patients. Paclitaxel concentrations were also determined on cycle 2, days 2 and 9 for patients randomized to placebo for cycle 1, who then crossed over to mifepristone for cycle 2. In addition to a baseline paclitaxel concentration prior to nab-paclitaxel administration (on day 1 and day 8), concentrations were measured 24 and 28 h after nab-paclitaxel administration on days 2 and 9. Because mifepristone can inhibit its own metabolism, mifepristone trough concentrations were measured on plasma sampled on days 1 and 8 of cycle 1 (just prior to day 1 and 8 mifepristone dose administration). For paclitaxel sample collection, 7 ml of whole blood were placed in a heparin vacutainer and centrifuged at 2000 rpm for 15 min. Plasma was transferred to a cryovial and stored at −80 °C. Whole blood for mifepristone levels was drawn in 7 ml heparin vacutainers, inverted gently 10 times then immediately centrifuged at 2000×*g* for 15 min with 1 ml of plasma aliquoted to cryotubes then frozen upright at −20 °C. Paclitaxel concentration assays were performed by The University of Chicago Pharmacology Core Facility, and mifepristone concentration assays were performed by Microconstants, Inc (La Jolla, CA).

### Correlative studies

To verify GR blockade, levels of both ACTH and cortisol were drawn at baseline prior to the initiation of mifepristone/placebo and nab-paclitaxel, and on day 1 of cycle 1, after a single dose of mifepristone. For patients randomized to placebo for cycle 1, both ACTH and cortisol were drawn on day 1 of cycle 2 as well. Because there are diurnal variations in ACTH and serum cortisol levels, both samples were obtained between 8 am and 10 am.

Unstained sections (3–5 microns in thickness) of primary tumor or metastatic tumor or both were requested for each patient enrolled on study. GR and androgen receptor (AR) nuclear expression were determined retrospectively via immunohistochemical (IHC) examination of archival tissue obtained for all patients enrolled. For determination of GR expression, anti-GR XP antibody (Cell Signaling, D8H2, 1:40 dilution) was used, and for determination of AR expression, anti-AR antibody (DAKO, AR441, 1:300 dilution) was used. Staining with these antibodies was performed according to methods previously published by Belova et al. (Belova et al. 2009). Tumors were considered GR or AR positive if greater than 10 % of cancer cells stained positively for nuclear GR or AR, respectively.

### Statistical analysis

This was a randomized, double-blinded, phase I, dose-escalation clinical trial of mifepristone versus placebo with nab-paclitaxel for metastatic or locally advanced breast cancer patients. For each dose level, 5 patients were randomized to nab-paclitaxel plus mifepristone versus nab-paclitaxel plus placebo in a 3:2 ratio for the first cycle of treatment. Patients were unblinded at the end of cycle 1 and crossed over to mifepristone beginning in cycle 2. Dose-escalation proceeded according to the standard ‘3 + 3’ design among patients randomized to mifepristone.

Pharmacokinetic samples were collected in order to determine whether the addition of mifepristone alters the pharmacokinetics of nab-paclitaxel. Serum samples for pharmacokinetic analyses were collected from all patients at baseline; cycle 1, day 2 (C1D2); cycle 1, day 9 (C1D9); and additional samples were collected from patients randomized to placebo on cycle 2 day 2 (C2D2); and cycle 2, day 9 (C2D9). Due to the small number of patients enrolled, pharmacokinetic data were summarized using graphical methods. We used the Wilcoxon rank sum test to compare continuous variables between groups. Linear regression models were used to determine the relationship between continuous measures; normality assumption was checked and variables were transformed if necessary.

## Results

### Patient characteristics

A total of nine women were enrolled between December of 2011 and March of 2013. The median age of participants was 56 years (range 47–74). Clinical and response characteristics are shown in Table 1. Of the nine patients enrolled, all but one had received prior taxane-based chemotherapy, seven in the adjuvant setting, and one in both the adjuvant and metastatic disease settings. However, only one patient was taxane-refractory, defined as recurrence or progression within 6 months of prior taxane exposure. Eight of the nine patients had relapsed after adjuvant therapy, and one presented with de novo metastatic disease.

**Table 1:** Clinical characteristics and response

Patient	Cycle 1 randomization	# Prior Txs for MBC	Prior taxane^a^	Taxane refractory^b^	Best response
Dose level 1: nab-paclitaxel 100 mg/m^2^ + mifepristone 300 mg					
1	Placebo	0	N	N/A	PR
2	Mifepristone	0	Y	N	PD
3	Placebo	2	Y	N	PD
4	Mifepristone	2	Y	N	CR
Dose level −1: nab-paclitaxel 80 mg/m^2^ + mifepristone 300 mg					
5	Mifepristone	0	Y	N	PR
6	Mifepristone	0	Y	Y	SD^c^
7	Placebo	0	Y	N	CR
8	Placebo	3	Y	N	PR
9	Mifepristone	3	Y	N	PD

*PR* partial response, *PD* progressive disease, *CR* complete response, *SD* stable disease, *MBC* metastatic breast cancer, *Txs* treatments, *n/a* not available, *Y* yes, *N* no

^a^In the adjuvant setting, with exception of patient 4 who received taxane in both adjuvant and metastatic disease setting

^b^Defined as recurrence or progression within 6 months of taxane exposure

^c^Unconfirmed response, all other responses confirmed

### Dose-limiting toxicities

Because of the potential for cumulative toxicity from nab-paclitaxel, only those patients randomized to mifepristone for cycle 1 were used for DLT determination (see Table 2). Of the 4 patients treated at dose level 1, patients 2 and 4 received mifepristone cycle 1 and were evaluable for DLT. Both patients experienced dose-limiting neutropenia. Patient 2 discontinued study treatment after cycle 2 for rapid progression of disease. Patient 4 remained on study treatment for 4 cycles with nab-paclitaxel dose reduction and filgrastim growth factor support, but discontinued treatment after four cycles for persistent neutropenia.

**Table 2:** Dose limiting toxicities in patients randomized to mifepristone for cycle 1

Patient	DLT	Type of DLT
Dose level 1: nab-paclitaxel 100 mg/m^2^ + mifepristone 300 mg		
2-M	Y	Neutropenia
4-M	Y	Neutropenia
Dose Level −1: nab-paclitaxel 80 mg/m^2^ + mifepristone 300 mg		
5-M	Y	Neutropenia
6-M	N	None
9-M	Y	Neutropenia

Only patients randomized to mifepristone for cycle 1 were used for DLT determination

M denotes patients randomized to mifepristone for cycle 1

*Y* yes, *N* no

Because two DLTs were observed at dose level 1, the starting dose of nab-paclitaxel was reduced from 100 to 80 mg/m^2^ for dose level −1 (again, given weekly for 3 weeks in a row followed by a 1 week break). The mifepristone dose was continued at 300 mg/d the day prior to and the morning prior to nab-paclitaxel administration. Of the 5 patients treated at dose level −1, patients 5, 6, and 9 received mifepristone for cycle 1 and were evaluable for DLT. Patients 5 and 9 both experienced a dose-limiting neutropenia. Patient 6 did not experience a DLT. Patient 5 was able to continue on therapy for 10 cycles with a dose reduction of the nab-paclitaxel to 60 mg/m^2^ and the initiation of growth factor support. She ultimately came off of study after 10 cycles for neuropathy, and continued on single agent nab-paclitaxel for an additional 19 cycles at a further reduced dose and a modified schedule. Patient 6 received 4 cycles of therapy and ultimately came off of study after four cycles for PD. After cycle 2, her nab-paclitaxel dose was reduced to 60 mg/m^2^ secondary to neuropathy. Patient 9 was found to have PD after 2 cycles.

Common grade 1 toxicities observed on the study included fatigue, nausea, vomiting, constipation, diarrhea, anorexia, rash, and arthralgias. The only grade 2 toxicities observed were alopecia and sensory neuropathy.

The majority of patients who received growth factor support received 2–3 days of filgrastim, with rapid recovery of their counts. However, due to the DLT of neutropenia observed at both nab-paclitaxel dose levels (100 and 80 mg/m^2^), and the possibility that the neutropenic DLTs were a result of a pharmacokinetic drug–drug interaction between nab-paclitaxel and mifepristone leading to increased plasma paclitaxel concentrations, a decision was made to close the study and proceed with pharmacokinetic analysis.

To explore the possibility of a pharmacodynamic (PD) interaction between mifepristone and nab-paclitaxel, we looked at the relationship between the log-fold reduction in the absolute neutrophil count (ANC) during cycle 1 and the randomized treatment. We found that patients randomized to mifepristone for cycle 1 had a greater log-fold reduction in ANC as compared to those who were randomized to placebo (see Additional file 1: Figure S1A, p = 0.05). A similar analysis of log-fold reduction in ANC and nab-paclitaxel dose level (100 vs. 80 mg/m^2^), showed no dose effect (see Additional file 1: Figure S1B, p = 0.46). We also used a linear regression model to determine the relationship between log-fold ANC reduction and 24-h paclitaxel concentration, and found that ANC decreases by approximately 5.5 % for a 10 ng/mL increase in paclitaxel concentration (p = 0.04). Although limited by the small sample size, we did not find that this relationship was different between placebo and mifepristone randomized patients (p = 0.94, paclitaxel concentration by treatment interaction). Similarly, no interaction was found between paclitaxel concentration and dose groups (p = 0.87).

### Tumor and response characteristics

Of the nine patients treated on study overall, two patients had a CR, three a PR, one SD, and three PD. Four patients were treated at dose level 1 (100 mg/m^2^ of nab-paclitaxel + mifepristone 300 mg), and five at dose level −1 (80 mg/m^2^ of nab-paclitaxel + mifepristone 300 mg). Of the four patients treated at dose level 1, one had a complete response (CR), one had a partial response (PR), and two had progression of disease (PD). Of the five patients treated at dose level −1, one had a CR, two had a PR, one had stable disease (SD, unconfirmed), and one had PD. As outlined in Table 3, while five of the study participants had estrogen receptor (ER) positive disease at the time of initial diagnosis, all but one of the patients had triple-negative disease at the time of recurrence (patient 3 had ER-positive disease at initial diagnosis, and did not undergo a biopsy of a metastatic site at the time of recurrence).

**Table 3:** Tumor and response characteristics

Patient	ER 1°	PR 1°	ER met	PR met	GR^a^	AR^a^	Best Response	# of cycles	Reason for discontinuation
1-P	n/a	n/a	−	−	+	−	PR	6	Neuropathy
2-M	+	+	−	−	−	wk+	PD	2	PD
3-P	+	+	n/a	n/a	−	−	PD	2	PD
4-M	+	+	−	−	+	wk+	CR	4	Neutropenia
5-M	−	−	−	−	+	−	PR	10	Neuropathy
6-M	+	−	−	−	+	−	SD^b^	4	PD
7-P	−	−	−	−	+	−	CR	9	PD
8-P	−	−	−	−	−	+	PR	5	Neuropathy
9-M	+	+	−	−	wk+	+	PD	2	PD

P denotes patients randomized to placebo for cycle 1 with cross over to mifepristone for cycle 2

M denotes patients randomized to mifepristone for cycle 1

*ER* estrogen receptor, *PR* progesterone, receptor, *AR* androgen receptor, *GR* glucocorticoid receptor, *1*° primary, *met* metastatic, *wk* weak, *n/a* not available

^a^Performed on both primary and metastatic tumor (when available) and were concordant

^b^Radiographically unconfirmed

**Fig. 1:**
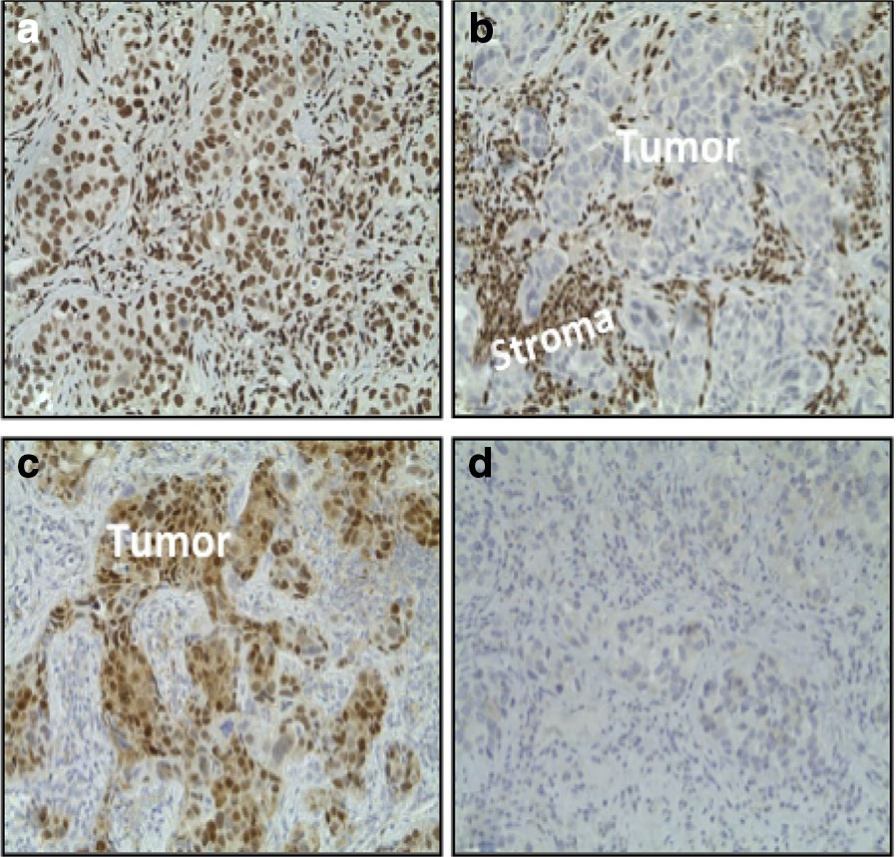
Immunohistochemical staining for GR and AR in primary breast tumors. **a** Tumor cells staining positively for GR. **b** tumor cells staining negative for GR (note that stromal cells stain positively for GR). **c** Tumor cells staining positive for AR. **d** Tumor cells staining negative for AR. *AR* androgen receptor, *GR* glucocorticoid receptor. **a**, **d** Tumors from patient 7. **b**, **c** Tumors from patient 8

Glucocorticoid receptor (GR) and androgen receptor (AR) expression was determined using immunohistochemical staining (see Fig. 1). As mifepristone is not only a strong GR and PR antagonist, but also a weak AR antagonist, we also wanted to determine AR expression of the tumors and perform an exploratory correlation of expression of both GR and AR to response. GR and AR staining was performed on both the primary tumor and the recurrence (when available). For the seven tumor pairs where both the primary tumor and the recurrent tumor were available (patient 1 presented with de novo metastatic disease, and patient 3 did not undergo a biopsy of a metastatic site at the time she developed disease recurrence), there was concordance between the primary and the recurrent tumors for both GR and AR. Six tumors were GR-positive (five strongly positive, one weakly positive). Of the five patients with strongly GR-positive tumors, two had a CR, 2 had a PR, and one had SD (unconfirmed). One additional patient had a tumor that was weakly GR-positive; she experienced PD after two cycles of study therapy (patient 9). Four tumors were positive for AR; two were strongly positive and two were weakly positive. Of the two patients with strongly AR-positive tumors, one had a PR and one had PD. No tumor was strongly positive for both GR and AR.

Of the four patients who had TNBC at the time of initial diagnosis, all responded to therapy (one had a CR, and three had PRs). Of these four patients, three had tumors that were GR-positive/AR-negative, and one had a tumor which was AR-positive/GR-negative.

### Pharmacokinetics

**Fig. 2:**
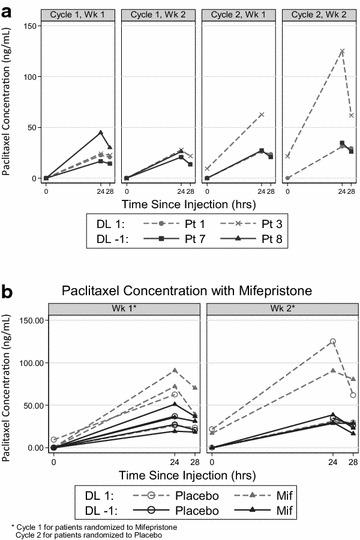
Plasma paclitaxel concentrations at 24 and 28 h. **a** Patients 1, 3, 7, and 8 were randomized to placebo/nab-paclitaxel during C1. Patients 1, 7, and 8 had no significant increase in paclitaxel levels when treated with mifepristone/nab-paclitaxel in C2. However, patient 3 had higher paclitaxel levels in C2 when treated with mifepristone/nab-paclitaxel. **b** patients treated at dose level 1 (nab-paclitaxel 100 mg/m^2^) had higher plasma paclitaxel concentrations than those patients treated at dose level −1 (nab-paclitaxel 80 mg/m^2^). *C1* cycle 1, *C2* cycle 2

Plasma concentrations of paclitaxel for all patients are shown in Additional file 2: Table S1, and plasma mifepristone (and its active metabolites) concentrations are shown in Additional file 2: Table S2. For patient 1, who received placebo for cycle 1 and then went on to receive mifepristone for cycle 2, paclitaxel concentrations were similar in both cycles. For patient 3, however, paclitaxel concentrations were higher when mifepristone was administered with nab-paclitaxel compared to when nab-paclitaxel was given with placebo. Figure 2a shows paclitaxel concentrations over time for all patients who received placebo for cycle 1 and then went on to receive mifepristone for cycle 2. Patient 3 was the only patient with significantly higher plasma paclitaxel concentrations when mifepristone was administered with nab-paclitaxel as compared to nab-paclitaxel alone. When evaluating paclitaxel concentrations over time by dose level (Fig. 2b), when nab-paclitaxel was administered with mifepristone, the patients with the three highest concentrations were treated at dose level 1 (100 mg/m^2^ of nab-paclitaxel), while those who received 80 mg/m^2^ had lower plasma paclitaxel concentrations.

Plasma mifepristone concentrations, as well as its active metabolites, were evaluated on days 1 and 8 for cycle 1 in all patients, and during cycle 2 in those patients randomized to placebo for cycle 1. For those patients receiving placebo for cycle 1, plasma mifepristone levels were undetectable, as expected (see Additional file 2: Table S2). No consistent increase of mifepristone levels were seen in the second cycle relative to the first. The concentration of mifepristone (C-1073) and its active metabolites (RU42633, RU42698, and RU42848) were higher on day 1 than 8 in some patients, and lower in others.

### Analysis of cortisol and ACTH

Serum cortisol and ACTH were evaluated at baseline and again 24 h after a single dose of mifepristone 300 mg or placebo on day 1 of cycle 1. For those randomized to placebo for cycle 1, cortisol and ACTH values were also determined on day 1 of cycle 2. When placebo was administered for cycle 1, cortisol and ACTH levels remained unchanged. After a single dose of mifepristone, serum cortisol levels increased 2–3 fold (Additional file 1: Figure S2A), demonstrating effective GR inhibition. ACTH levels did not increase uniformly: for some patients ACTH levels remained relatively unchanged, and in others, they increased 2–3 fold, suggesting that the peak ACTH response in some individuals had occurred prior to the 24 h serum collection (Additional file 1: Figure S2B).

## Discussion

The primary objective of this study was to determine the safety and tolerability of the combination of the GR steroidal antagonist mifepristone and nab-paclitaxel. We also sought to confirm that systemic GR antagonism was occurring with the mifepristone dose used in this trial. Dose-limiting neutropenia (as specified in the protocol) was observed at both doses of nab-paclitaxel; therefore, we halted the study and analyzed the pharmacokinetic data for a potential drug–drug interaction. The novel randomized design we used allowed us the opportunity to understand how mifepristone impacted nab-paclitaxel clearance in the same patient, as those patients who were randomized to placebo for cycle 1 crossed over to receive mifepristone for cycle 2. Data from the pharmacokinetic studies suggest that for some patients, there appeared to be a delay in paclitaxel clearance when co-administered with mifepristone (see Fig. 2a, b). For the remaining patients, there was no clear evidence of a drug–drug interaction, and paclitaxel concentrations 24 h after administration were consistent with levels observed in initial phase I studies of nab-paclitaxel (Nyman et al. 2005; Ando et al. 2012).

The neutropenia observed in this phase I trial, while dose-limiting by protocol definition, was safe and manageable, and the majority of patients who experienced neutropenia continued to receive study treatment with dose delays, dose modifications, and/or the institution of growth factor support. Most of the five patients who received growth factor support received 2–3 days of once daily filgrastim, with rapid recovery of their counts. Of the nine patients studied, four did not experience neutropenia, and thus did not require dose modifications or growth factor support. Of the five who did experience neutropenia, one discontinued study treatment due to persistent neutropenia despite growth factor support and dose modification (patient 4), with the other four remaining on study therapy with the assistance of growth factor support. Of note, patient 4 continued to have neutropenia on nab-paclitaxel even after the discontinuation of mifepristone, and had to discontinue nab-paclitaxel therapy completely due to persistent neutropenia despite dose reduction and the institution of growth factor support. Of the other four, only one also required a dose reduction for the management of neutropenia (patient 5), but she was able to stay on study therapy for 10 cycles, before coming off for neuropathy.

To explore if there was a pharmacodynamic interaction between mifepristone and nab-paclitaxel (leading to a decreased neutrophil count), we compared the log-fold reduction in ANC for those randomized to mifepristone for the first cycle of therapy to those randomized to placebo. While we did see a greater log-fold reduction in ANC when mifepristone was given compared to placebo, this correlation was borderline significant. However, some cases of neutropenia observed in this trial did not appear to be the result of delayed nab-paclitaxel clearance. The greater log-fold reduction seen when mifepristone was administered with nab-paclitaxel as compared to placebo is suggestive of a pharmacodynamic interaction between mifepristone and nab-paclitaxel leading to neutropenia.

We hypothesize that some cases of neutropenia observed in this study may be related to the enhancement of chemotherapy-induced neutrophil apoptosis in the setting of a GR antagonist, and not delayed clearance of nab-paclitaxel. Paclitaxel levels observed in this trial were consistent with those levels observed in other trials using similar doses of nab-paclitaxel, further suggesting a pharmacodynamic mechanism for the neutropenia, rather than a simple pharmacokinetic increase in paclitaxel levels. Glucocorticoids have been shown to protect neutrophils from apoptosis (Kato et al. 1995). In the setting of GR antagonists, it is possible that neutrophils will be more susceptible to cell death, particularly in the setting of apoptosis-inducing cytotoxic therapies such as nab-paclitaxel. As such, combining GR antagonism with chemotherapy in general may require growth factor support. As some patients did not develop neutropenia, it is likely that dose modifications and growth factor support will need to be individualized based on observed toxicities.

In an exploratory analysis evaluating tumor GR and AR expression, we observed that tumors with strong GR expression were typically AR-negative, and tumors with strong AR expression were typically GR-negative. Expression of BR is negatively regulated by AR signaling in prostate cancer (Xie et al. 2015), and our data suggest that this this is likely the case in breast cancer as well. Six patients had tumors that were GR-positive (five were strongly GR-positive, and one was weakly GR-positive), and all six of these tumors were triple-negative at the time of recurrence (two of these patients initially presented with ER-positive disease, but tumors had converted to TNBC at the time of disease recurrence). Interestingly, of the five patients with GR strongly-positive tumors, four patients had a response to therapy (two CRs and two PRs, all confirmed), and one had SD (unconfirmed). The patient with GR weakly-positive disease progressed rapidly. These observational data support our hypothesis that GR-positive TNBC may benefit from the addition of a GR antagonist to chemotherapy.

In conclusion, nab-paclitaxel plus mifepristone appears to be a tolerable regimen, with a primary toxicity of neutropenia. While neutropenia was a protocol-defined DLT, the combination of mifepristone and nab-paclitaxel at both dose levels studied was tolerable, and the neutropenia was easily managed. It also appears that the interaction between mifepristone and nab-paclitaxel is not necessarily pharmacokinetic in nature, but rather pharmacodynamic. Thus, given the ease of management of the neutropenia and the promising efficacy of the combination, we are proceeding with a randomized phase II trial of nab-paclitaxel with mifepristone versus placebo in patients with advanced, GR positive, triple-negative breast cancer. We will use a nab-paclitaxel dose of 100 mg/m^2^ given 3 weeks on followed by 1 week off, with a mifepristone dose of 300 mg given the day before and the day of each nab-paclitaxel dose. As neutropenia was not observed in all patients, even at the higher dose level used, we will use the standard dose of 100 mg/m^2^, the standard dose used in the metastatic setting.

## Additional files

10.1186/s40064-016-2457-1. Log-fold reduction in absolute neutrophil count by mifepristone versus placebo or by dose level. A, in general, patients administered mifepristone/nab-paclitaxel had a greater reduction in absolute neutrophil count (ANC) compared to placebo/nab-paclitaxel (p = 0.05). The gray solid line represents the average linear regression of log-fold reduction in ANC for patients receiving placebo/nab-paclitaxel. The solid black line represents the average linear regression of log-fold reduction in ANC for patients receiving mifepristone/nab-paclitaxel. The dashed line represents the averaged linear regression for all patients. B, there was no significant difference in ANC reduction between patients who received nab-paclitaxel dose level 1 (100 mg/m^2^) compared to dose level -1 [nab-paclitaxel 80 mg/m^2^ (p = 0.46)]. The gray solid line represents the linear regression of log-fold reduction in ANC in patients receiving nab-paclitaxel 100 mg/m^2^. The solid black line represents the average linear regression of log-fold reduction in ANC in patients receiving nab-paclitaxel 80 mg/m^2^. The dashed line represents the averaged linear regression for all patients. Reg, regression lines. ANC, absolute neutrophil count. **Figure S2**: Serum cortisol and ACTH levels before therapy initiation and 24 h after the first dose of mifepristone 300 mg (n = 9) 1. A, serum cortisol levels increased by 2–3 fold in every patient. B, ACTH increases were variable. DL, dose level.
10.1186/s40064-016-2457-1. Plasma paclitaxel concentrations (ng/mL)^**^. **Table S2**: Concentrations of mifepristone and its active metabolites (ng/mL).
