# Supplementary figures and images for: A randomized phase I trial of nanoparticle albumin-bound paclitaxel with or without mifepristone for advanced breast cancer
Source: Springerplus. 2016 Jun 30;5(1):947. doi: 10.1186/s40064-016-2457-1 (PMC4929099; doi:10.1186/s40064-016-2457-1)

## Slide 1
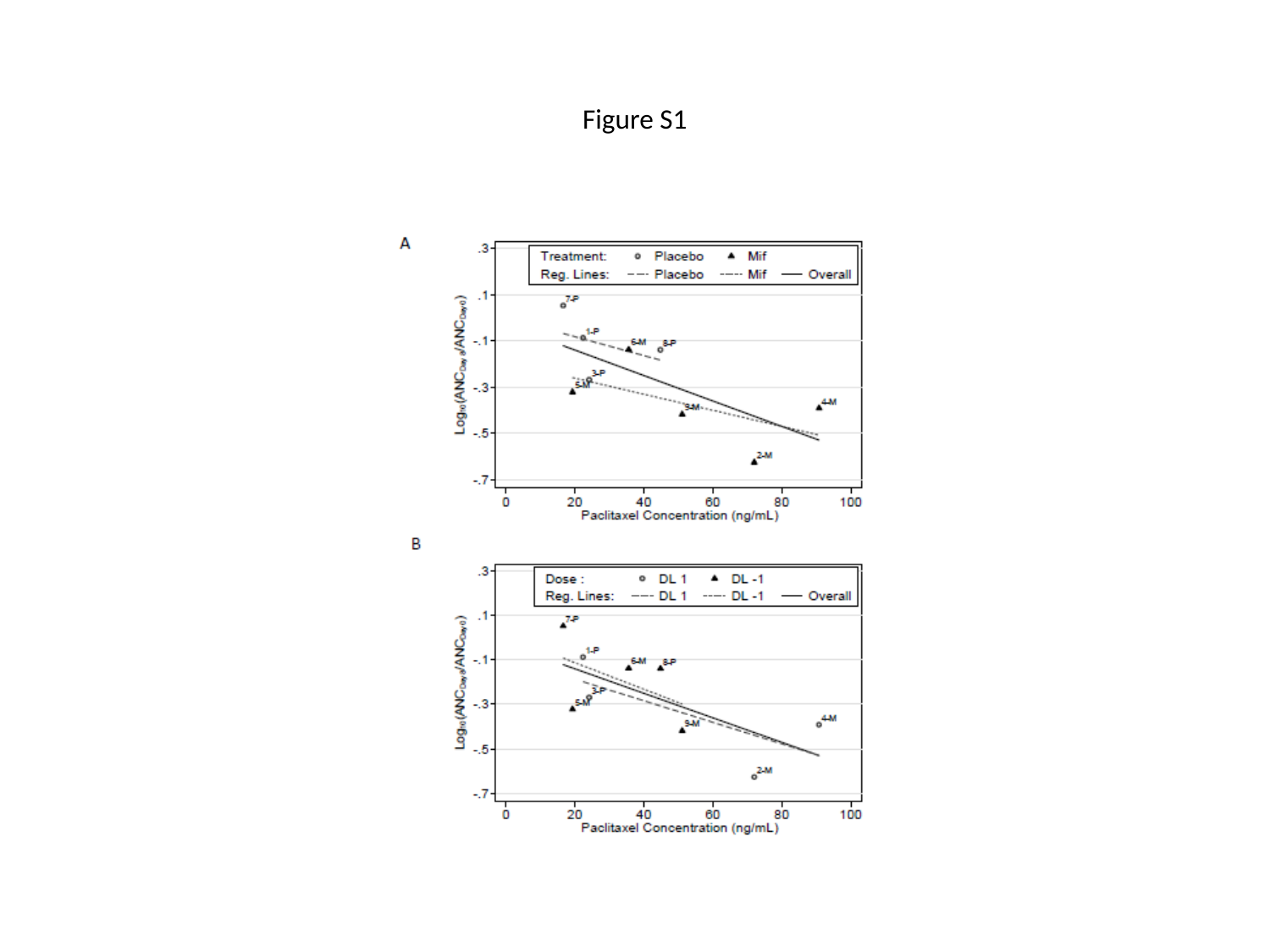

# Figure S1

## Slide 2
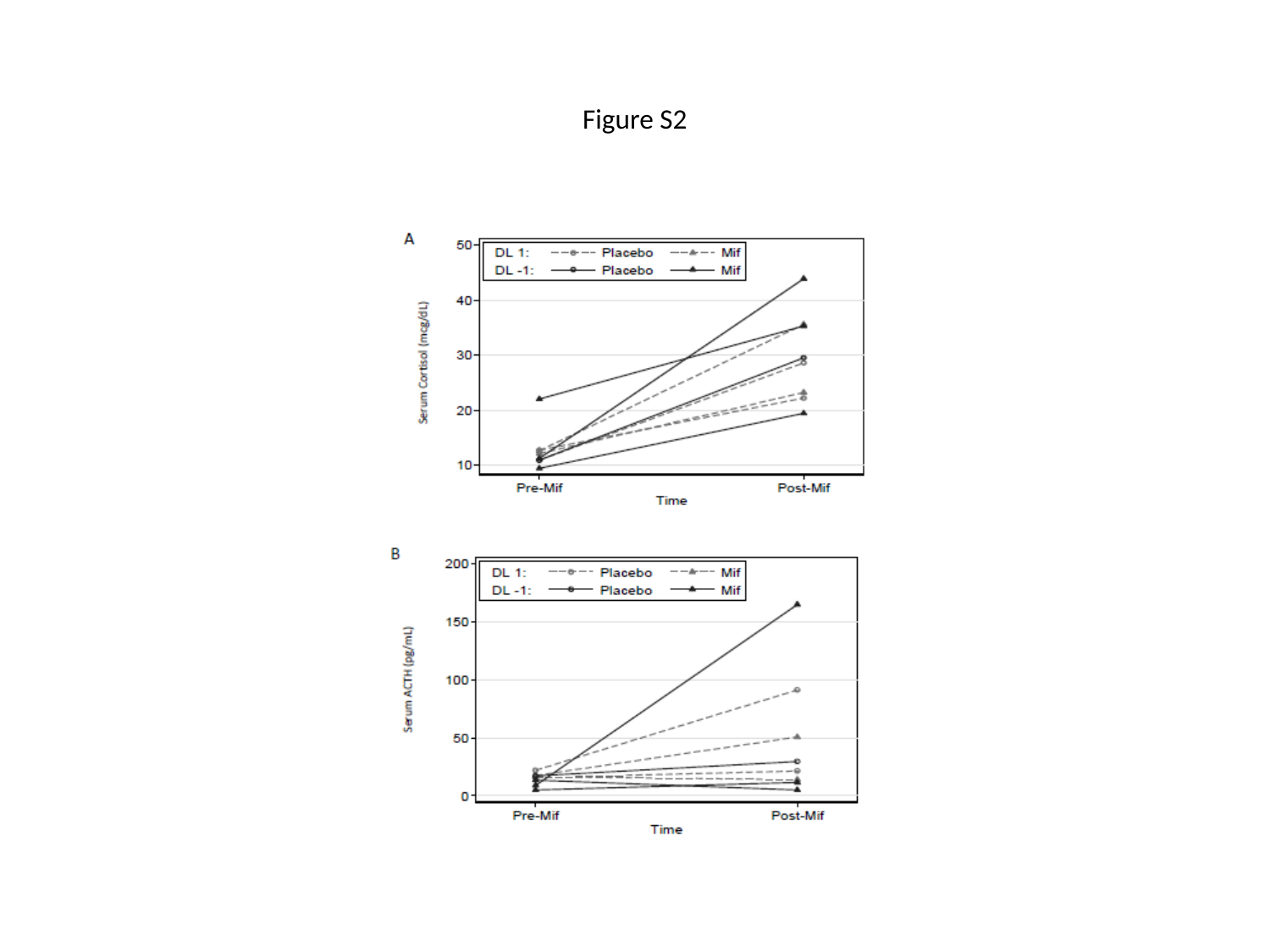

# Figure S2

Supplement: Supplementary file 1 — 10.1186/s40064-016-2457-1. Log-fold reduction in absolute neutrophil count by mifepristone versus placebo or by dose level. A, in general, patients administered mifepristone/nab-paclitaxel had a greater reduction in absolute neutrophil count (ANC) compared to placebo/nab-paclitaxel (p = 0.05). The gray solid line represents the average linear regression of log-fold reduction in ANC for patients receiving placebo/nab-paclitaxel. The solid black line represents the average linear regression of log-fold reduction in ANC for patients receiving mifepristone/nab-paclitaxel. The dashed line represents the averaged linear regression for all patients. B, there was no significant difference in ANC reduction between patients who received nab-paclitaxel dose level 1 (100 mg/m2) compared to dose level -1 [nab-paclitaxel 80 mg/m2 (p = 0.46)]. The gray solid line represents the linear regression of log-fold reduction in ANC in patients receiving nab-paclitaxel 100 mg/m2. The solid black line represents the average linear regression of log-fold reduction in ANC in patients receiving nab-paclitaxel 80 mg/m2. The dashed line represents the averaged linear regression for all patients. Reg, regression lines. ANC, absolute neutrophil count. Figure S2: Serum cortisol and ACTH levels before therapy initiation and 24 h after the first dose of mifepristone 300 mg (n = 9) 1. A, serum cortisol levels increased by 2–3 fold in every patient. B, ACTH increases were variable. DL, dose level. [file 40064_2016_2457_MOESM1_ESM.pptx]
