# Supplementary material for: A randomized phase I trial of nanoparticle albumin-bound paclitaxel with or without mifepristone for advanced breast cancer
Source: Springerplus. 2016 Jun 30;5(1):947. doi: 10.1186/s40064-016-2457-1 (PMC4929099; doi:10.1186/s40064-016-2457-1)
Supplement: Supplementary file 2 — 10.1186/s40064-016-2457-1. Plasma paclitaxel concentrations (ng/mL)**. Table S2: Concentrations of mifepristone and its active metabolites (ng/mL). [file 40064_2016_2457_MOESM2_ESM.docx]

**Table S1: Plasma paclitaxel concentrations (ng/mL)****

|  | **Cycle 1** | | | | | | **Cycle 2** | | | | | | |
| --- | --- | --- | --- | --- | --- | --- | --- | --- | --- | --- | --- | --- | --- |
|  | **Cycle 1 Day 1/2** | | | **Cycle 1 Day 8/9** | | | **Cycle 2 Day 1/2** | | | | **Cycle 2 Day 8/9** | | |
| **Pt*** | **Pre-Rx** | **24 h** | **28 h** | **C1D8** | **24 h** | **28 h** | **C2D1** | **24 h** | **28 h** | | **C2D8** | **24 h** | **28 h** |
| **Dose Level 1: nab-paclitaxel 100 mg/m2 + mifepristone 300 mg** | | | | | | | | | | | | | |
| **1-P** | 0 | 22.4 | 20.8 | 0 | ND | ND | 0 | 26.1 | 23.4 | 0 | | 31 | 29.3 |
| **2-M** | 0 | 72 | 39.1 | 0 | ND | ND |  |  |  |  | |  |  |
| **3-P** | 0 | 24.2 | 22.6 | 0 | 27.7 | 21.7 | 9.3 | 62.6 | ND | 21.6 | | 125.3 | 61.7 |
| **4-M** | 0 | 90.8 | 70.2 | 16.9 | 90.5 | 80.5 |  |  |  |  | |  |  |
| **Dose Level -1: nab-paclitaxel 80 mg/m2 + mifepristone 300 mg** | | | | | | | | | | | | | |
| **5-M** | 0 | 19.3 | 18.4 | 0 | 29.8 | 16.5 |  |  |  |  | |  |  |
| **6-M** | 0 | 35.6 | 31.2 | 0 | 38.6 | 23.3 |  |  |  |  | |  |  |
| **7-P** | 0 | 16.6 | 14.3 | 0 | 20.6 | 13.7 | 0 | 27.2 | 20.6 | ND | | 38.4 | 26.1 |
| **8-P** | 0 | 44.8 | 30 | 0 | 26.2 | ND | 0 | 37.1 | ND | ND | | ND | ND |
| **9-M** | 0 | 51.1 | 36.8 | 0 | 28.8 | 29 |  |  |  |  | |  |  |

**Table S2: Concentrations of mifepristone and its active metabolites (ng/mL)**

*P denotes patients randomized to placebo for cycle 1 with cross over to mifepristone for cycle 2. M denotes patients randomized to mifepristone for cycle 1.

**C1D1=cycle 1, day 1; C1D8=cycle 2, day 2; etc; Pt, patient; ND, not done; Rx, treatment

|  | **Cycle 1 Day 1** | | | | **Cycle 1 Day 8** | | | | **Cycle 2 Day 1** | | | | **Cycle 2 Day 8** | | | |
| --- | --- | --- | --- | --- | --- | --- | --- | --- | --- | --- | --- | --- | --- | --- | --- | --- |
| **Pt** | **Mifepristone** | **RU42633** | **RU42698** | **RU42848** | **Mifepristone** | **RU42633** | **RU42698** | **RU42848** | **Mifepristone** | **RU42633** | **RU42698** | **RU42848** | **Mifepristone** | **RU42633** | **RU42698** | **RU42848** |
| **Dose Level 1: nab-paclitaxel 100 mg/m^2^ + mifepristone 300 mg** | | | | | | | | | | | | | | | | |
| **1-P** | 0 | 0 | 0 | 0 | 0 | 0 | 0 | 0 | 954 | 1240 | 338 | 342 | 1870 | 2730 | 589 | 727 |
| **2-M** | 1480 | 1880 | 685 | 822 | ND | ND | ND | ND |  |  |  |  |  |  |  |  |
| **3-P** | 0 | 0 | 0 | 0 | 0 | 0 | 0 | 0 | 1480 | 2550 | 455 | 1670 | 4070 | 3710 | 803 | 1520 |
| **4-M** | 1370 | 1770 | 442 | 1150 | 1230 | 1510 | 519 | 931 |  |  |  |  |  |  |  |  |
| **Dose Level -1: nab-paclitaxel 100 mg/m^2^ + mifepristone 300 mg** | | | | | | | | | | | | | | | | |
| **5-M** | 2770 | 2600 | 837 | 426 | 1310 | 1700 | 531 | 632 |  |  |  |  |  |  |  |  |
| **6-M** | 1140 | 988 | 235 | 184 | 1100 | 1680 | 318 | 561 |  |  |  |  |  |  |  |  |
| **7-P** | 0 | 0 | 0 | 0 | 0 | 0 | 0 | 0 | 1680 | 3120 | 529 | 1100 | 1400 | 2540 | 486 | 1040 |
| **8-P** | 0 | 0 | 0 | 0 | 0 | 0 | 0 | 0 | 835 | 1270 | 289 | 456 | 1950 | 1820 | 477 | 555 |
| **9-M** | 2310 | 3100 | 680 | 555 | 4280 | 5000 | 773 | 928 |  |  |  |  |  |  |  |  |

*P denotes patients randomized to placebo for cycle 1 with cross over to mifepristone for cycle 2. M denotes patients randomized to mifepristone for cycle 1.

**C1D1=cycle 1, day 1; C1D8=cycle 2, day 2; etc; Pt, patient; ND, not done
